# Prevalence of Community Perinatal Psychiatrists in the US

**DOI:** 10.1001/jamanetworkopen.2024.26465

**Published:** 2024-08-07

**Authors:** Amanda Koire, Mariella Suleiman, Polina Teslyar, Cindy H. Liu

**Affiliations:** 1 Department of Psychiatry, Brigham and Women’s Hospital, Boston, Massachusetts; 2 Harvard Medical School, Boston, Massachusetts; 3 Department of Psychiatry, Maimonides Medical Center, Brooklyn, New York; 4 Now with Department of Psychiatry, Icahn School of Medicine at Mount Sinai, New York, New York; 5 Department of Pediatric Newborn Medicine, Brigham and Women’s Hospital, Boston, Massachusetts

## Abstract

This cross-sectional study identifies and quantifies state-level shortages in community-based perinatal psychiatry care in the US.

## Introduction

In the US, 86% of women will become mothers and approximately 20% will experience a peripartum mood episode.^1^ Despite perinatal mental health accounting for substantial maternal mortality, 3 in 4 women do not receive treatment.^2^ One challenge for patients is finding psychiatric treatment. Nonpsychiatrist physicians have historically expressed a lack of training and confidence regarding mental health treatment of pregnant individuals, and many psychiatrists express similar hesitation. Meanwhile, long wait times for evaluation worsen depression and increase risk of self-harm ideation.^3^ Access to perinatal psychiatry subspecialists in academic centers is limited,^4^ yet the landscape of access in the community has not been well studied. This cross-sectional study identified and quantified state-level shortages in community-based perinatal psychiatry care.

## Methods

Perinatal psychiatrist deficit by state was estimated using the equation:

Deficit = [(0.22 × Annual State Births) – (No. of Perinatal Psychiatrists × Patient Panel Size)]/Patient Panel Size

Number of perinatal psychiatrists was defined as psychiatrists indicating “pregnancy, prenatal, postpartum” as an issue they treat on Psychology Today as of November 11, 2022. Births per state were obtained from US Census data. The equation incorporates the point prevalence of positive postpartum depression screening results during the first postpartum year^1^ and existing estimates of community psychiatry patient panel size range.^5^ Density of perinatal psychiatrists was defined as the number per 5000 births in the state. Google Trends provided the relative predominance by state of the search term *postpartum depression* from November 11, 2021, to November 11, 2022; abortion restrictiveness as of November 15, 2022, was defined by the Guttmacher Institute. Spearman correlations were performed among variables of care density, postpartum depression searches, and abortion restrictiveness. Hypothesis tests were 2-sided (α = .05). Data analysis was performed using Prism, version 9.3.1, software. Exemption of institutional review board review was granted by Mass General Brigham. Informed consent was not required because the study used publicly accessible deidentified data. eMethods in Supplement 1 provides additional details. This study followed the STROBE reporting guidelines. Data on race were not available in the dataset, and data on ethnicity were not collected to mimic a maximally flexible search.

## Results

Twenty-one states had a ratio of less than 1 psychiatrist offering peripartum treatment per 5000 births (Figure). South Dakota, Montana, and Mississippi demonstrated the lowest density, while New York, Connecticut, and Rhode Island demonstrated the highest (Table); many states were estimated to require dozens to hundreds of additional specialists to fully meet projected needs. Where the density of perinatal psychiatrists was lower, Google searches for *postpartum depression* were more prevalent (*R* = −0.49; *P* < .001). More restrictive state policies on abortion were correlated with lower density of perinatal psychiatrists (*R* = −0.56; *P* < .001) and higher prevalence of Google searches (*R* = 0.55; *P* < .001).

**Figure.**
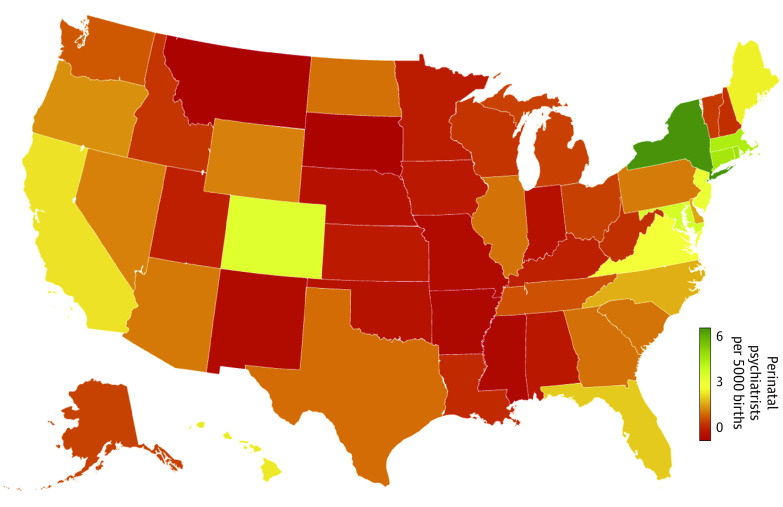
Heatmap of Perinatal Psychiatrist Density in the US Data are based on the number of psychiatrists on the Psychology Today website indicating “pregnancy, prenatal, postpartum” as an issue they treat.

**Table.** Perinatal Psychiatrist Density in the US

State	Annual births in 2020	Perinatal psychiatrists per 5000 births	Estimated deficit of perinatal psychiatrists
Montana	10 791	0.00	5-24
South Dakota	10 960	0.00	5-24
Mississippi	35 473	0.14	15-77
Arkansas	35 251	0.14	14-76
Missouri	69 285	0.22	22-144
New Mexico	21 903	0.23	9-51
Indiana	78 616	0.38	30-168
Nebraska	24 291	0.41	9-51
Oklahoma	47 623	0.42	17-101
Alabama	57 647	0.52	21-123
Iowa	36 114	0.55	12-75
Kansas	34 376	0.58	9-70
Minnesota	63 443	0.63	21-133
Utah	45 702	0.66	13-94
Kentucky	51 668	0.68	15-106
Louisiana	57 328	0.78	17-118
New Hampshire	11 791	0.85	3-24
West Virginia	17 323	0.87	5-35
Wisconsin	60 594	0.91	19-125
Idaho	21 533	0.93	8-46
Vermont	5133	0.97	1-10
Ohio	129 191	1.04	30-257
Alaska	9469	1.06	2-19
Michigan	104 074	1.06	21-204
Tennessee	78 689	1.21	16-154
Washington	83 086	1.26	15-161
Texas	368 190	1.44	30-678
Georgia	122 473	1.47	8-223
North Dakota	10 059	1.49	3-21
Illinois	133 298	1.50	18-252
South Carolina	55 704	1.53	8-106
Arizona	76 947	1.56	0-135
Pennsylvania	130 693	1.57	11-241
Wyoming	6128	1.63	2-12
Nevada	33 653	1.63	7-66
Oregon	39 820	1.76	7-77
Delaware	10 392	1.92	1-19
North Carolina	116 730	2.01	18-224
Florida	209 671	2.27	0-360
California	420 259	2.47	0-671
Hawaii	15 785	2.53	0-28
Maine	11 539	2.60	1-21
Virginia	94 749	2.85	0-160
New Jersey	97 954	3.16	0-141
Colorado	61 494	3.33	0-103
Maryland	68 554	3.57	0-100
Massachusetts	66 428	4.22	0-100
Connecticut	33 460	4.33	0-43
Rhode Island	10 101	4.46	0-14
New York	209 338	5.92	0-194

## Discussion

When attempting to self-refer in the community, individuals in most states confront a shortage of psychiatrists advertising peripartum care. Herein, we assumed optimal circumstances regarding insurance coverage and psychiatrist appointment availability and did not restrict the search to psychiatrists with additional formal training or credentials; however, prospective patients would encounter barriers to accessing care corresponding to a deficit of hundreds to thousands of perinatal psychiatrists nationwide. One study limitation is that no approach can be comprehensive in assessing the psychiatrist workforce, and some patients may receive treatment from obstetricians or nonphysician health care professionals. However, the data reflect what perinatal patients may encounter when seeking a psychiatrist. States with restrictive abortion policies are likely to see higher levels of psychological distress and suicidality^6^ in the peripartum population yet are underserved with regards to community access to perinatal psychiatry. Potential solutions for inadequate and unequal access to evidence-based perinatal psychiatric care include improving education regarding mental health treatment during pregnancy for general psychiatrists and obstetricians and minimizing geographic constraints on access to treatment.

## Notes

Supplement 1. **eMethods.** Acquisition and Analysis of Data
eReferences.

Supplement 2.
Data Sharing Statement
