# Supplementary material for: Prevalence of Community Perinatal Psychiatrists in the US
Source: JAMA Netw Open. 2024 Aug 7;7(8):e2426465. doi: 10.1001/jamanetworkopen.2024.26465 (PMC11307129; doi:10.1001/jamanetworkopen.2024.26465)
Supplement: Supplement 1. — eMethods. Acquisition and Analysis of Data eReferences. [file jamanetwopen-e2426465-s001.pdf]

Supplementary Online Content

Koire A, Suleiman M, Teslyar P, Liu CH. Prevalence of community perinatal psychiatrists in the US. *JAMA Netw Open*. 2024;7(8):e2426465. doi:10.1001/jamanetworkopen.2024.26465

eMethods. Acquisition and Analysis of Data

eReferences.

This supplementary material has been provided by the authors to give readers additional information about their work.

© 2024 Koire A et al. *JAMA Network Open*.

eMethods. Acquisition and Analysis of Data

This study analyzed publicly accessible data from multiple sources data as follows. Using custom programming scripts for web data extraction, the number of MD/DO psychiatrists searchable on Psychology Today[1] (a database widely used by the lay public) as of 11/11/2022 who indicated 'pregnancy, prenatal, postpartum' as an 'Issue' they are willing to treat was identified for each state. While other databases regarding the psychiatrist workforce exist, the selected database is frequently used by patients, focuses on community psychiatrists, and specifically provides information regarding whether the psychiatrists self-identify as treating peripartum individuals. The number of births in each state was obtained from the 2020 Census[2]. The perinatal psychiatrist deficit equation projects anticipated need for services by combining point prevalence of positive postpartum depression screening during the first year postpartum[3] with the annual births in the state. The equation then accounts for the existing number of psychiatrists in the state, operating under the assumption that their panels have been filled by these cases, and then estimates how many additional psychiatrists would be needed to meet remaining demand. The reported range corresponds to theoretical patient panel sizes of 100 and 500 patients per psychiatrist based on existing literature regarding community psychiatry patient panel sizes[4]. Google Trends[5] provided the relative predominance by state of the search term 'postpartum depression' for a one year period between 11/11/2021-11/11/2022, with values calculated on a scale ranging between 0-100 such that a higher value indicates a higher proportion of all search queries in that state. Degree of abortion restrictiveness as of 11/15/2022 was defined by the Guttmacher Institute[6] which attaches to each state a designation ranging from 'most protective' to 'most restrictive'; these designations were converted to a scale from 1-7, respectively, for statistical analysis. Density of perinatal psychiatry care was calculated using the above data as the number of perinatal psychiatrists per 5,000 births, with 5,000 births corresponding to approximately 1,000 cases of perinatal depression.

eReferences.

1. Psychology Today. Find a Psychiatrist. https://www.psychologytoday.com/us/psychiatrists?search=

2. U.S. Census Bureau. State Population by Characteristics: 2010-2020. Annual Estimates of the Civilian Population by Single Year of Age and Sex for the United States and States: April 1, 2010 to July 1, 2020. Accessed November 11, 2022. https://www.census.gov/programs-surveys/popest/technical-documentation/research/evaluation-estimates/2020-evaluation-estimates/2010s-state-detail.html

3. Wisner KL, Sit DKY, McShea MC, et al. Onset Timing, Thoughts of Self-harm, and Diagnoses in Postpartum Women With Screen-Positive Depression Findings. *JAMA Psychiatry*. 2013;70(5):490. doi:10.1001/jamapsychiatry.2013.87

4. McQuistion HL, Zinns R. Workloads in Clinical Psychiatry: Another Way. *Psychiatr Serv*. 2019;70(10):963-966. doi:10.1176/appi.ps.201900125

5. Google Trends. Postpartum Depression. https://trends.google.com/trends/?geo=US

© 2024 Koire A et al. *JAMA Network Open*.

6. Guttmacher Institute. Interactive Map: US Abortion Policies and Accesss After Roe. Accessed November 15, 2022. https://states.guttmacher.org/policies/?gclid=CjwKCAiAheacBhB8EiwAItVO26AomBr0lomm6EozPNNOfWrN4AeKs0lSjkmt4cpPgztOa61qKUPm4xoCvKAQAvD_BwE

© 2024 Koire A et al. *JAMA Network Open*.
