# Supplementary material for: Prevalence of Community Perinatal Psychiatrists in the US
Source: JAMA Netw Open. 2024 Aug 7;7(8):e2426465. doi: 10.1001/jamanetworkopen.2024.26465 (PMC11307129; doi:10.1001/jamanetworkopen.2024.26465)
Supplement: Supplement 2. — Data Sharing Statement [file jamanetwopen-e2426465-s002.pdf]

# Data Sharing Statement

Koire. Prevalence of Community Perinatal Psychiatrists in the US. *JAMA Netw Open*.
Published August 07, 2024. doi:10.1001/jamanetworkopen.2024.26465

**Data**
**Data available:** Yes
**Data types:** Data (not involving human participants), Data dictionary
**How to access data:** akoire@bwh.harvard.edu
**When available:** With publication

**Supporting Documents**
**Document types:** None

**Additional Information**
**Who can access the data:** Anyone requesting the data
**Types of analyses:** Any purpose
**Mechanisms of data availability:** Without investigator support
